# Supplementary material for: Multifaceted barriers associated with clinical breast examination in sub-Saharan Africa: A multilevel analytical approach
Source: PLoS One. 2025 Jan 13;20(1):e0316800. doi: 10.1371/journal.pone.0316800 (PMC11730425; doi:10.1371/journal.pone.0316800)
Supplement: Attachment — Submitted filename: Response to reviwers comments.docx [file pone.0316800.s002.docx]

**PONE-D-24-26002**

**Multifaceted barriers impacting clinical breast examination in sub-Saharan Africa: A multilevel analytical approach**

Dear Editor,

We are grateful to you and the reviewers for your comments on our paper. We would also take this opportunity to thank the reviewers for finding merit in this paper and suggesting some revisions. We have taken notice of all the comments raised by the reviewers and have responded accordingly as follows. Please note that the reviewers' comments are in black whereas our responses are in red, and have been highlighted accordingly in the revised manuscript.

I have one major concern. As outlined in the DHS documentation, specifically the 'Guide to DHS Statistics,' DHS data are generated using a complex sampling design that includes both cluster and stratified sampling, along with sampling probabilities. When using DHS data involving multiple levels (e.g., household, census enumeration areas, national level), which I believe applies to the data used in this manuscript, it is essential to incorporate not only sample weights but also the primary sampling unit (PSU) variable and stratification variable in the analyses. However, these important analytical details are not mentioned. I assume they were not incorporated into the analyses presented in the manuscript. (In response to this comment, the authors may need more time than usual for revisions. If this is the case, please contact PLOS ONE to request an extension.)

Response: We agree with you. This was taken into consideration in analyzing our study which has been specified in the methods “The complex sampling design of the DHS survey was accounted for by using the "*svyset*" command in Stata (which takes into account the primary sampling units (PSU) and the stratification of the clusters). Kindly refer to pp.7. Thank you.

It’s unclear why only Burkina Faso, Cote d’Ivoire, Ghana and Kenya were selected if the study findings are intended to be generalized across all SSA countries. The manuscript states that these four countries were selected "based on the defined timeframe, context, and availability of variables of interest" (Page 3). What exactly is the defined timeframe? What specific variables of interest were unavailable in the datasets from other SSA countries?

Response: Thank you for this comment. At the initial stage of analyzing the study, only these four countries had a variable that measures Clinical Breast Examination (CBE). However, an additional three countries (Mozambique, Senegal and Tanzania) have published a recent DHS dataset that measures CBE. Hence the analysis has been revised to include these countries making seven. Overall, these seven countries were selected based on the availability of the outcome variable in the most recent (within the last five years) Demographic and Health Surveys (DHS) dataset.

How to justify that the study findings from those 4 countries are generalizable to the broader SSA region?

Response: Thank you for your invaluable contribution. This limitation has been acknowledged which reads “Moreover, the study included only seven SSA countries that have available DHS datasets with a variable measuring CBE in the individual recode file (IR file), which makes us limited to extrapolating our results to all SSA countries”.

The manuscript also lacks sufficient details about the studied barriers, i.e. permission, money, distance, and traveling alone. What does ‘permission to access healthcare’ really refer to? Which survey datasets were used to obtain each of these variables? The justification provided ("Informed by previous studies [34, 35, 36]") is too vague and shallow.

Response: Thank you for this comment. These four barrier variables are captured by the DHS dataset measuring difficulties accessing healthcare facilities. These include difficulty in getting permission to access healthcare, distance to the nearest health facility, getting money needed for treatment, and travelling alone. All these barrier variables have a binary response as “a big problem=1” and “not a big problem=0” as captured in the DHS dataset”. Hence, our decision to select these exposures was informed by their availability in the dataset, as well as their inclusion in existing studies.

In the revision, please create a supplemental file detailing which specific DHS data files were used for acquiring these key study variables, as well as other variables. Additionally, I suggest providing the programming codes used for the modeling analysis to allow readers to reproduce the results.

Response: The study used the individual recode data (IR file) of the DHS dataset of respective countries with an available variable that measures CBE. We have ensured that a detailed description of the analytical procedure is provided in the method which is enough for others to replicate the study, instead of sharing our programming codes. Thank you.

Some expressions in the manuscript are vague or confusing. For example, “… to implement comprehensive strategies aimed at empowering women.” The phrase "empowering women" seems to suggest more than just reducing the barriers discussed in the paper.

Response: Thank you for the comment. We have now removed this sentence.

Please add more information to the tables, such as the sample size (N) for columns, whether percentages are row or column percentages, add in a table footnote the specific analytical approaches used to generate the results in each table.

Response: Thank you. A footnote has been provided under Table 1-3 indicating that all percentages are calculated in row percentages.

2. Your ethics statement should only appear in the Methods section of your manuscript. If your ethics statement is written in any section besides the Methods, please move it to the Methods section and delete it from any other section. Please ensure that your ethics statement is included in your manuscript, as the ethics statement entered into the online submission form will not be published alongside your manuscript.

Response: Thank you. This concern has been addressed. Kindly refer to pp.8.

3. Please upload a copy of Supplementary File 1 to which you refer in your text on page 22. Please amend the file type to 'Supporting Information'. If the Supplementary file is no longer to be included as part of the submission please remove all reference to it within the text. [Note: HTML markup is below. Please do not edit.]

Response: We have uploaded the supplementary file.

Reviewer #1:

Thank you for the opportunity to review the manuscript titled “Multifaceted barriers impacting clinical breast examination in sub-Saharan Africa: A multilevel analytical approach.”
This is well written manuscript and good work was done by the authors
I have some suggestions for the authors to consider

Results
• Page 9: “However, women who faced travel alone barrier were 1.13 times more likely to utilise CBE compared to those who were not.” The authors should provide the confidence interval in support of the odds

Response: Thank you. The confidence interval has been added “However, women who faced travel-alone barriers were 1.19 times (95%CI: 1.10-1.28) more likely to utilise CBE compared to those who did not face travel-alone as a barrier” Please refer to pp.10.

• “Women who were using modern or traditional methods of contraceptives were 1.18 and 1.31 times more likely to undergo CBE than those who were not using any form of contraceptives.” The authors should provide the confidence interval in support of the odds when reporting the odds in a signal phrase or as the integral of the text. Odds alone might not be enough in making some comparison of the outcome of variables, confidence interval plays a major role in that so try and provide that as a support to the odds

Response: This has been addressed “Women who were using modern or traditional methods of contraceptives were 1.20 (95%CI: 1.14-1.27) and 1.32(95%CI: 1.19-1.47) times more likely to undergo CBE than those who were not using any form of contraceptives” Please refer to pp.10.

Discussion
• First Paragraph: “Despite this modest improvement, it is crucial to acknowledge that the current uptake rates remain far below optimal levels.” When a comparison statement is made like this, it will be very crucial to state the comparators, therefore, I will urge the authors to state the optimal level of uptake
Response: Thank you for the comment. We have now addressed this.

Reviewer #2: Major

1. Potential Bias Due to Dropped Missing Values (n = 15,255).
The manuscript mentions that a substantial number of observations (n = 15,255) were excluded due to missing values. The exclusion of such a large portion of the sample may introduce significant bias into the study results. The authors should discuss the potential impact of these missing values on the findings. Specifically, they should consider whether the missing data is random or if it is systematically related to certain characteristics (e.g., socioeconomic status, location, education level) that could influence the outcome (CBE uptake). If the missing data is not completely random, it could skew the results, leading to biased estimates of the association between the exposure variables and CBE uptake.

Response: Thank you for drawing attention to this. The error has been corrected. Kindly refer to the flow chart (Figure 1) showing the sample size determination. We observed no missing values in the selected variables in the study, with the exception of “507 don’t know responses” which were dropped from the analysis.

2. Potential Bias Introduced by Dichotomized Measurements. The study utilizes dichotomized measurements for several key variables (e.g., barriers such as permission, financial constraints, distance, and traveling alone). While dichotomization simplifies the analysis, it may lead to a loss of information and potentially introduce bias. Dichotomizing variables can obscure important variations within the data and create artificial cut-off points that may not accurately reflect the true distribution or relationship of the variables in the population. For instance, treating 'distance' or 'financial constraints' as binary variables might oversimplify complex barriers that exist on a continuum. The authors should discuss these limitations in the manuscript and consider alternative approaches, such as using the original continuous or ordinal scales, which may provide a more nuanced understanding of the relationships between the variables and CBE uptake.

Response: Thank you for this insight. However, kindly be informed that these four variables were captured as a binary response category in the DHS dataset, thus whether the respondent is finding it difficult to access healthcare facilities or not based on these barriers.

3. Table 2 Fallacy: Misinterpretation of Exposure and Outcome Relationships
The use of a single model in Table 4 to estimate the effects of four exposures (permission, financial constraints, distance, and traveling alone) on one outcome (CBE uptake) with the same set of confounders adjusted for all exposures presents a potential issue known as the "Table 2 fallacy" (Westreich & Greenland, 2013). This approach may lead to misinterpretation of the associations due to the assumption that the same set of covariates confounds all exposures equally. Each exposure-outcome relationship should ideally be considered separately with its own directed acyclic graph (DAG) to identify the minimal sufficient adjustment set for each specific exposure. The authors are encouraged to revise the analytical strategy to reflect this. By identifying and adjusting for the correct set of covariates for each exposure separately, the authors can provide more accurate effect estimates and interpretations, thus strengthening the validity of their findings.

Response: Thank you for your comment. However, table 4 does not suffer from "Table 2 fallacy." It presents results from multilevel logistic regression models, showing how the adjusted odds ratios (aORs) of various barriers and community-level factors evolve across different models (from the null model to the full model). In effects, it highlights how each variable affects the outcome of interest as more explanatory variables are added. The progressive models and the model fit statistics (AIC, BIC, Log-likelihood) are included, allowing for a robust assessment of the impact of the variables. Therefore, the analysis considers the whole model evolution and outcomes, which mitigates the risk of “Table 2 fallacy”. Besides, the interpretation of the results is based on the full model.
