# Supplementary material for: Multifaceted barriers associated with clinical breast examination in sub-Saharan Africa: A multilevel analytical approach
Source: PLoS One. 2025 Jan 13;20(1):e0316800. doi: 10.1371/journal.pone.0316800 (PMC11730425; doi:10.1371/journal.pone.0316800)
Supplement: Attachment — Submitted filename: Response to Reviewers.docx [file pone.0316800.s003.docx]

PONE-D-24-26002R1

Multifaceted barriers impacting clinical breast examination in sub-Saharan Africa: A multilevel analytical approach

PLOS ONE

 01 November, 2024

Dear Editor,

On behalf of the coauthors of Manuscript ID PONE-D-24-26002R1, I thank you and the reviewers for reviewing our manuscript, “Multifaceted barriers impacting clinical breast examination in sub-Saharan Africa: A multilevel analytical approach”. We have addressed each point raised by the reviewers below and tracked any corresponding changes in the manuscript

Thanks for addressing the initial comments, particularly by clarifying analytical details. However, there remain major concerns in the analyses:

1. The DHS data provides sample weights within each country but does not include guidance for combining results across multiple countries. This suggests the analyses would be conducted separately by country, as shown in Tables 1 and 2, with the 'All countries' row removed. Logistic regressions, currently presented in Table 4, should also be fitted separately by country.

Response: Thank you for your contribution. Please be informed that pooling DHS data from multiple countries is a common and robust research practice aligned with DHS guidelines on sampling and weighting procedures across countries. This approach also helps account for the effect of country-specific variations on the association under study. Detailed procedures for pooling DHS data for multi-country analysis can be found in the *DHS Sampling and Household Listing Manual* (pp. 26–29), available at <https://dhsprogram.com/pubs/pdf/DHSM4/DHS6_Sampling_Manual_Sept2012_DHSM4.pdf>

2. In Table 4, it appears that Model IV is intended as the final working model. Presenting results from the other models that were not selected may be confusing, which echoes the comments from the other reviewer. It might also be helpful to emphasize they are adjusted association effects instead of casual effects per se.

Response: Thank you for the insight. Presenting the results for each model, along with the final model, enables readers to better understand how individual and contextual factors contribute to the associations examined in the study. We have clarified that, given the study's cross-sectional design, the models show associations rather than causality; this limitation is also discussed in the limitations section.

3. In the current model, 'Travel alone' does not show a statistically significant association with CBE uptake, while it does show significance in the marginal associations in Table 2 for most countries. This discrepancy may result from correlations among covariates in the final model. It’s understandable that 'Distance' and 'Travel alone' might be correlated. Then it’s a question whether the model selection procedure has completed. Consider performing backward or stepwise selection to refine the model, potentially retaining only one of 'Distance' or 'Travel alone' in the final model for correct finding and interpretation.

Response: Thank you. Please note that we assessed multicollinearity among the main predictors, as well as the individual and contextual variables, using the variance inflation factor (VIF) before fitting the regression models. The assessment yielded a mean VIF of 4.49, indicating no significant collinearity issues. Additionally, we would like to highlight that in the current model, "Travel alone" is statistically significant, with an adjusted odds ratio (aOR)=1.19; 95%CI: 1.10–1.28.

Additional minor comments:

1. The authors included random effects in the models to account for cluster effects. Could you clarify what these clusters represent? Do they represent regions within a country? So are they the random effect nested in each country?

Response: In our analysis, clusters represent enumeration areas (EAs) as per the DHS data structure. These clusters are indeed nested within each country and account for intracluster correlations, addressing potential biases from the hierarchical data structure.

2. There are hypothesis tests for random effects. They provide direct evidence of including random effects or not in model approaches.

Response: In this study, random effects were included to account for the hierarchical structure of the data, where individual women were nested within clusters (enumeration areas (EAs)). To evaluate the significance of these random effects, we used the Intra-Class Correlation Coefficient (ICC) and Proportional Change in Variance (PCV). We appreciate your invaluable contribution.

3. On page 9, in the first sentence of the first paragraph, the country name should be "Mozambique" instead of "Cote d'Ivoire" in the context: "with only 5.5% (p=0.014)...a big problem in Cote d'Ivoire." Please check the text thoroughly for accuracy in all details.

Response: This error has been corrected. It reads “In Burkina Faso, Cote d’Ivoire, Ghana and Mozambique, difficulty in getting permission to visit health facilities emerged as a significant barrier, with only 5.5% (p=0.014) utilising CBE among women who considered it a big problem in Mozambique”. Thank you.

Reviewer #2: Table 2 Fallacy: Misinterpretation of Exposure and Outcome Relationships

The manuscript presents four models in Table 4, which aim to estimate the effects of different factors on Clinical Breast Examination (CBE) uptake:
• Model 1: CBE uptake ~ Permission + Money + Distance + Travel alone
• Model 2: CBE uptake ~ Age + Education + Occupational status + Religion + Health facility visit (last 12 months) + Wealth index + Contraceptive usage + Media exposure (within a week)
• Model 3: CBE uptake ~ Type of residence + Country
• Model 4: CBE uptake ~ All variables
The authors estimate the causal effects of four specific factors on CBE uptake (Permission, Money, Distance, and Travel alone) by calculating adjusted odds ratios (aORs) in the full model (Model 4). While Model 4 uses criteria such as AIC, BIC, and Log-likelihood for model selection, it implicitly assumes that each of these four main factors is confounded by all other factors in the model, including other variables from Models 1, 2, and 3. This approach may lead to what is commonly known as the "Table 2 fallacy."
The "Table 2 fallacy," introduced by Westreich and Greenland (2013), occurs when multiple effect estimates are presented from a single model and mistakenly interpreted as causal effects. It involves assuming that each variable's relationship with the outcome is equally interpretable as causal, which can be misleading, especially when variables have distinct roles or relationships with the outcome.
To improve the analysis and avoid this Table 2 fallacy, I recommend the following steps to justify the confounding adjustment set:
1. Separate Tables for Each Exposure: Present each exposure's effect estimates and potential confounders in individual tables to reduce confusion.
2. Use of Causal Diagrams (DAGs): Employ directed acyclic graphs (DAGs) to clarify the causal relationships and identify which variables should be adjusted for in each model. Analyze each exposure variable separately, using a unique DAG for each, to determine the minimal sufficient adjustment set. This ensures that each model includes only the confounders relevant to that specific exposure-outcome relationship.
3. Tailored Confounder Sets: Refine the confounder set for each exposure variable to prevent over-adjustment or adjustment for irrelevant variables. Additionally, consider the following adjustments:
By taking these steps, you can better address the potential for misinterpretation and provide more robust conclusions on the factors affecting CBE uptake.

Response: Thank you for your insightful contribution to this study. First and foremost, please note that this study relied on cross-sectional data, hence in effect, it does not establish any causality but rather an association between barrier variables (Permission + Money + Distance + Travel alone) and CBE uptake accounting for both individual and contextual level factors using multilevel logistic regression model instead of the traditional logistic regression model due to the hierarchical structure of the data, where individual women were nested within clusters (enumeration areas (EAs)). We would like to reemphasize that Table 4’s presentation, with Models I-IV, does not fall under the "Table 2 fallacy" as described by Westreich and Greenland, because it does not present each variable’s association with the outcome in isolation as if each relationship were causal. Instead, it shows a progression of models that incrementally adjust for various levels of covariates— both individual and contextual—reflecting an analytical framework to examine associations rather than causal inferences.
