# Supplementary material for: Multifaceted barriers associated with clinical breast examination in sub-Saharan Africa: A multilevel analytical approach
Source: PLoS One. 2025 Jan 13;20(1):e0316800. doi: 10.1371/journal.pone.0316800 (PMC11730425; doi:10.1371/journal.pone.0316800)
Supplement: Attachment — Submitted filename: pone.0316800.docx [file pone.0316800.s004.docx]

**Multifaceted barriers associated with clinical breast examination in sub-Saharan Africa: A multilevel analytical approach**

Castro Ayebeng, ^1,2^ Joshua Okyere,^2,3*^ Christiana Okantey^4^, and Isaac Yeboah Addo^5,6^

1. School of Demography, Australian National University, Canberra, ACT, Australia
2. Department of Population and Health, University of Cape Coast, Cape Coast, Ghana.
3. School of Human and Health Sciences, University of Huddersfield, Queensgate, Huddersfield, England, United Kingdom
4. Department of Adult Health, School of Nursing and Midwifery, University of Cape Coast, Cape Coast, Ghana
5. Concord Clinical School, University of Sydney, Sydney, Australia
6. Centre for Social Research in Health, The University of New South Wales, Sydney, Australia

**Corresponding author***

**Emails:**

CA: [castro.ayebeng@stu.ucc.edu.gh](mailto:castro.ayebeng@stu.ucc.edu.gh)

JO: [joshuaokyere54@gmail.com](mailto:joshuaokyere54@gmail.com)/joshua.okyere@hud.ac.uk

CO: [christiana.okantey@ucc.edu.gh](mailto:christiana.okantey@ucc.edu.gh)

IYA: [i.addo@unsw.edu.au](mailto:i.addo@unsw.edu.au) or yebaddo9@yahoo.com

**KEY MESSAGES BOX**

**Key Findings:**

- The uptake of clinical breast examination (CBE) in seven sub-Saharan African countries (Burkina Faso, Cote d’Ivoire, Ghana, Kenya, Mozambique, Senegal and Tanzania) is notably low, with a prevalence of only 19.2%[95%CI:18.5-19.8].
- Significant barriers to CBE uptake include difficulty in getting permission, financial constraints, and distance to access healthcare facilities.
- Interestingly, women who reported travel-alone barriers were more likely to utilise CBE than those who did not encounter this barrier.

**Implications:**

- Addressing barriers such as financial constraints, difficulty in getting permission, and distance to access healthcare facilities is crucial for improving CBE uptake.
- Empowerment strategies focusing on financial independence and self-governance are essential.
- Policies should also aim to mitigate physical or spatial barriers to healthcare access.

**Recommendations:**

- Implement comprehensive strategies that empower women financially and enhance their autonomy.
- Improve infrastructure and healthcare accessibility to reduce physical and spatial barriers.
- Foster community and healthcare system support to address the identified barriers effectively, thereby increasing CBE utilisation rates in sub-Saharan Africa.

**Abstract**

**Objectives**: Clinical breast examination (CBE) open the pathway to early detection and diagnosis of breast cancer. This study examined barriers to CBE uptake in seven sub-Saharan African (SSA) countries.

**Methods**: Data from the most current Demographic and Health Surveys of Burkina Faso, Cote d’Ivoire, Ghana, and Kenya Mozambique, Senegal and Tanzania was used. A weighted sample size of 65,486 women aged 25-49 years was used to estimate the pooled prevalence of CBE. We employed a multilevel logistic regression modelling technique, with results presented in adjusted odds ratios (aOR) along with a 95% confidence interval (CI).

**Results**: The pooled prevalence of CBE uptake in the studied SSA countries is low at 19.2% [95%CI: 18.5-19.8]. Screening uptake was significantly low among women reporting difficulty in getting permission (aOR=0.88, 95% CI: 0.82-0.95), and distance (aOR=0.95, 95% CI: 0.89-0.99), as well as those who reported financial constraints (aOR=0.92, 95% CI: 0.88-0.97), as barriers to access healthcare facilities. However, surprisingly, women who faced travel-alone barriers were 1.19 times (95%CI: 1.10-1.28) more likely to utilise CBE than those who did not face this barrier.

**Conclusions**: We conclude that barriers such as difficulties in obtaining permission, long distances to healthcare facilities, and financial constraints significantly reduce the likelihood of women undergoing CBE. The study underscores a need to improve access to healthcare facilities. Practically, this can be achieved by expanding mobile health services and integrating CBE into primary healthcare will help overcome distance-related challenges. Additionally, targeted outreach and transportation initiatives are necessary to support women facing travel barriers.

**Keywords**: Clinical Breast Examination; Screening; Public Health; Health-seeking behavior

**Background**

Breast cancer is a significant public health challenge globally, particularly in low- and middle-income countries (LMICs) where late-stage diagnosis and limited access to treatment contribute to high mortality rates [1,2]. Breast cancer screening is crucial for early detection, and allows for less aggressive treatment, increased survival rates, and a better quality of life for patients [3,4]. Breast cancer screening can also assist in detecting precancerous changes in the body, enabling preventive measures to reduce the risk of developing invasive breast cancer, and as a result, reducing treatment costs as treating early-stage cancer is generally less expensive than treating advanced-stage cancer [5,6,7]. Beyond individual benefits, breast cancer screening contributes to public health efforts by reducing the overall burden of breast cancer and improving population health outcomes, providing women with proactive control over their breast health [8].

A variety of methods can be employed in breast cancer screening with the objective of early detection of the disease. One common approach is mammography, which involves X-ray imaging of the breast tissues [9]. Mammography is widely known to be particularly effective for women over 40 years and is considered the gold standard for detecting breast cancer at early, more treatable stages [10,11]. Breast self-examination (BSE) is another screening method that involves women examining their own breasts regularly to detect any changes or abnormalities [12]. While BSE is simple and cost-effective, its effectiveness in reducing breast cancer mortality is debated, and it may lead to unnecessary anxieties if the result is a false positive [13,14]. Other methods, such as breast ultrasound and magnetic resonance imaging (MRI), are sometimes used in specific cases, such as for women with dense breast tissues or those at high risk of breast cancer [15,16]. Essentially, the choice of screening method depends on various factors, including age, risk factors, and resource availability; and a combination of methods may be used for optimal screening outcomes [17]. Another significant screening method warranting attention is the clinical breast examination (CBE). CBE is a cost-effective screening method recommended for early detection of breast cancer, especially in settings with limited resources and often involves a healthcare professional manually examining the breasts for lumps or other abnormalities [18,19]. Nevertheless, the utility of CBE in high-income countries such as the United States is currently subject to debate due to the widespread presence and advancements in mammography technology in such countries [20].

In Sub-Saharan Africa (SSA) where resources for breast screening are constrained despite the significant risk and burden of breast cancer, CBE is regarded as a crucial screening method for breast cancer prevention and early intervention. A recent cancer mapping study involving all 54 African countries revealed that breast cancer is the most predominant malignancy among females in the continent, surpassing 34 other identified cancer types, with a total of 186,598 new cases and 85,787 fatalities recorded solely in the year 2020 [21]. However, the prevalence of CBE utilisation and the barriers to its uptake in SSA, remain poorly understood. Thus, there is a significant dearth of studies in SSA that have focused on examining the prevalence of CBE uptake and identifying barriers to its utilisation. The lack of research can be attributed to various factors, such as resource constraints, with limited funding, infrastructure, and trained personnel restricting large-scale studies [22,23,24,25]. Additionally, the extensive prioritisation of infectious disease control, such as malaria and HIV over non-communicable diseases like breast cancer diverts research efforts and funding away from breast cancer screening behaviour studies [26,27,28,29,30]. Moreover, the lack of comprehensive health data in cancer registries and health information systems in several African countries poses additional challenges for designing population-based studies on CBE [24,31]. By pooling data from seven SSA countries (Burkina Faso, Cote d’Ivoire, Ghana, Kenya, Mozambique, Senegal and Tanzania) where variables related to CBE are available in the Demographic and Health Surveys (DHS) dataset, this study aimed to examine the status of CBE utilisation and identified common barriers that hinder its uptake. Findings from this study are essential for policymakers, healthcare providers, and researchers working to improve breast cancer screening and early detection programs in SSA.

**Methods**

**Data source and design**

This is a secondary cross-sectional analysis from seven SSA countries (Burkina Faso (2021), Cote d’Ivoire (2021), Ghana (2022), Kenya (2022), Mozambique (2022-23), Senegal (2023) and Tanzania (2022)) where variables related to CBE are available in the most recent (within the last five years) Demographic and Health Surveys (DHS) dataset. The DHS data is a nationally representative data derived from two-stage stratified sampling procedure in selecting research participants of households in low- and middle-income countries (LMICs). In the first phase, clusters/enumeration areas (EAs) are selected, guided by a sample frame developed during the preceding census of the respective countries, while in the second stage, a sample of households is drawn from each selected EA. Within selected households, women between the ages of 15 and 49 years were asked to provide information regarding their households, personal details, and their children, as well as some health screening behaviours such as screening for clinical breast cancer which is the outcome of interest in this study. The surveys employed similar data collection methodologies and comparable questionnaires, enabling cross-country comparisons [32]. This study analysed a weighted sample of 65,486 women aged 25-49 years from the individual recode file (IR file). We excluded women aged 15-24 years due to the low risk of breast cancer in this population [33]. Detailed determination of the final sample size for this study is specified in Figure 1.

**Figure 1**: Flow chart of sample determination

**Variables and measures**

***Outcome variable***

Clinical breast examination (CBE) is the outcome of interest in this study. This was derived from the question, “Have you ever had your breast examined by a healthcare provider? The responses were dichotomised as “yes =1” or “no=0”.

***Exposure variables***

Informed by previous studies^34-36^, and in alignment with the DHS data four barriers were used as the main exposure variables in this study. These include difficulties in getting permission to access healthcare, distance to the nearest health facility, getting money needed for treatment, and travelling alone. All these barrier variables have a binary response as “a big problem=1” and “not a big problem=0” as captured in the DHS dataset. In addition to these barriers, other individual and community-level factors were identified and adjusted for in the analyses based on empirical literature [25,34,35,36].

The individual-level factors were age (25-29, 30-34, 35-39, 40-44, and 45-49), level of education (no formal education, basic, secondary and higher), employment status (working or not working), religion (Christianity, Islam, and other), visit health facility in the last 12 months (yes or no), wealth index (poorest, poorer, middle, richer and richest), contraceptive usage (not using, modern contraceptive, and traditional method), and media exposure. Media exposure is a composite variable derived from three sets of variables: frequency of reading newspapers or magazines; frequency of listening to the radio; and frequency of watching television. The responses were categorised as (not at all=0, less than once a week=1, at least once a week=2, and almost every day=3). We used the ‘egen’ command in STATA to generate the composite variable where those who did not listen to the radio, watch TV or read newspaper/magazine at all were categorised as “no media exposure”, while the remaining responses were categorised as having media exposure. The community-level factors were type of residence (rural or urban) and country of residence.

**Analytical procedure**

We conducted a descriptive analysis of the percentage of women screened for CBE across seven countries, presenting distributions across identified barriers with chi-square tests for associations. Due to the hierarchical data structure where women were nested within a cluster, we used multilevel logistic regression models. Four models were constructed: the null model, Model I with the four main barriers (permission, money, distance, traveling alone), Model II adjusted for individual factors (age, education, employment, religion, wealth, health facility visits, contraceptive use, media exposure), and Model III including community-level factors (residence, country). Model IV adjusted for the effects of the individual, and community-level factors on the association between barrier variables and the outcome of interest. Odds ratios with 95% confidence intervals were presented, and multicollinearity was assessed with a variance inflation factor (VIF) mean score of 4.49. Analyses were performed using Stata 17, applying sample weights. The complex sampling design of the DHS survey was accounted for by using the "*svyset*" command in Stata (which takes into account the primary sampling units (PSU) and the stratification of the clusters).

To apply sample weights in a pooled data analysis, the standard weight variable for the individual recode file (v005) was first de-normalized as follows: v005 × (total female population 15-49 in the country)/(total number of women 15-49 interviewed in the survey) and then re-normalized so that the pooled sample average is 1. This was significant because, according to the DHS sampling and household listing manual, the normalised weight is not valid for pooled data, even data pooled for women and men in the same survey, because the normalisation factor is country and sex-specific [37].

**Fixed and random effect estimates**

The analysis incorporated both fixed and random effects. In the fixed effect analysis, we examined a set of variables encompassing individual and contextual factors. The goodness of fit of the mixed effect models was assessed using the Log-likelihood (LL) test, Akaike’s Information Criterion (AIC) and Bayesian Information Criterion (BIC). The model characterised by the lowest value of the information criterion was chosen as the best model in the analysis. The random effect analysis was aimed to assess variations between clusters (EAs). We calculated two essential metrics to quantify these variations: Intra-class correlation coefficient (ICC) and the proportional change in variance (PCV). The ICC gauges the extent of variation within clusters, specifically among individuals within the same cluster. It was computed using the formula:

ICC = [V_A_/ (V_A_ + π2/3)] = V_A_/(V_A_ + 3.29). Here, V_A_ represents the estimated variance in each model [38].

To assess the overall variation attributed to individual and contextual factors in each model, we utilised the proportional change in variance (PCV), calculated as:

PCV = (V_A_ – V_B_)/V_A_. In this equation, V_A_ is the variance of the initial model, and V_B_ is the variance of the model with additional terms [38]. Our presentation of the results was guided by the Strengthening the Reporting of Observational Studies in Epidemiology (STROBE) guidelines (S1 Table) [39].

**Ethical approval and consent to participate**

Ethical clearance was not required for our study because we used a publicly available DHS dataset. However, the DHS reports that both written and verbal informed consent were obtained from all participants. We accessed the datasets from the DHS Program after completing the necessary registration and obtaining approval for their use. We adhered to all ethical guidelines relevant to the use of the secondary dataset in research publications.

**Results**

**Prevalence of CBE**

Overall, the pooled prevalence of CBE across all the studied countries was 19.2% (95%CI: 18.5-19.8) (Table 1). However, the prevalence of CBE uptake varied across countries with the highest uptake being reported in Burkina Faso 31.1% (95%CI: 29.1-33.1) and the least from Tanzania 7.3% (95%CI: 6.5-8.2).

[Insert Table 1]

**Distribution of clinical breast examination across identified barriers by country**

In Burkina Faso, Cote d’Ivoire, Ghana and Mozambique, difficulty in getting permission to visit health facilities emerged as a significant barrier, with only 5.5% (p=0.014) utilising CBE among women who considered it a big problem in Mozambique. Difficulties in getting money needed for treatment also posed a significant barrier across all countries (p < 0.001), with substantially lower proportions in Mozambique (5.6%) and Tanzania (5.5%) undergoing CBE among those who found it challenging. Distance to a health facility was another significant barrier (p < 0.001), with a significantly lower percentage of women who consider it a big problem in all countries screening for CBE, particularly in Tanzania (4.9%) and Mozambique (5.4%) (Table 2).

Additionally, travelling alone was identified as an important barrier in Cote d’Ivoire, Ghana, Kenya, Mozambique, and Tanzania, with a notably lower proportion of the respondents in Mozambique (8%) and Tanzania (6.2%) screening for CBE among those who considered travelling alone as a challenge. Overall, the presence of at least one of the barriers significantly influenced the percentage of women undergoing CBE across all countries (p < 0.001), with significantly lower proportions undergoing screening in Mozambique (6.9%) and Tanzania (5.6%).

[Insert Table 2]

**Proportion of CBE utilisation among women by selected covariates**

Table 3 shows the distribution of CBE utilization by selected covariates. CBE utilization was high among women aged 35-39 years 20.6%(95%CI: 19.6-21.7), those with higher education 38.5%(95%CI: 36.4-40.6), those currently employed 20.7%(95%CI: 20.0-21.5), had visited a health facility in the last 12 months 22.9%(95%CI: 22.1-23.7), were using modern 21.7%(95%CI: 20.9-22.7) or traditional contraceptive methods 24.6%(95%CI: 22.6-26.8), had media exposure 21.9%(95%CI: 21.2-22.6), and resided in the urban setting 24.5%(95%CI: 23.5-25.5).

[Insert Table 3]

**Barriers associated with CBE uptake among women in seven SSA countries**

Women reporting difficulty in getting permission (aOR=0.88, 95% CI: 0.82-0.95), and distance (aOR=0.95, 95% CI: 0.89-0.99), as well as those who reported financial constraints (aOR=0.92, 95%CI: 0.88-0.97) to access healthcare facilities, had lower odds of CBE uptake. However, surprisingly, women who faced travel-alone barriers were 1.19 times (95%CI: 1.10-1.28) more likely to utilise CBE compared to those who did not face this barrier.

Higher screening odds were observed among women aged 35-39 years (aOR=1.31, 95%CI: 1.22-1.40), Muslim (aOR=1.56, 95%CI: 1.34-1.80) and Christians (aOR=1.81, 95%CI: 1.57-2.09), and those from the richest households (aOR=2.01, 95%CI: 1.81-2.23). The odds of undergoing CBE were low among women with no formal education (aOR=0.37, 95%CI: 0.33-0.41) or having only primary education (aOR=0.43, 95%CI: 0.39-0.47), those who had no visitation to a health facility in the last 12 months (aOR=0.62, 95%CI: 0.59-0.66), and women with no media exposure (aOR=0.75, 95%CI: 0.70-0.80). Women who were using modern or traditional methods of contraceptives were 1.20 (95%CI: 1.14-1.27) and 1.32(95%CI: 1.19-1.47) times more likely to undergo CBE than those who were not using any form of contraceptives.

Additionally, community-level factors such as residence and country of residence exhibited substantial effect, with women resident in rural areas (aOR=0.88, 95%CI: 0.83-0.94), as well as all countries showing significantly reduced odds of CBE uptake compared to the reference country (Burkina Faso).

[Insert Table 4]

***Random effects results***

The random-effect models demonstrated that community-level variance slightly increased from 0.152 in the null model to 0.196 in the full model, indicating some variability in the outcome due to differences between communities. The proportional change in variance (PCV) indicates that 18.1% of the variation between communities is explained by the full model compared to the null model. The intra-class correlation coefficient (ICC) remains fairly consistent, ranging from 4.1% to 4.8%, suggesting that about 4-5% of the total variability in the outcome can be attributed to differences between communities across the models. This highlights the importance of accounting for community-level factors in understanding the variation in the outcome (see Table 4).

***Model fit statistics***

The model fitness statistics evaluate how well the multilevel logistic regression model fits the data. We employed the Loglikelihood (LL) test, Akaike’s Information Criterion (AIC), and Bayesian Information Criterion (BIC) for comparing different models. Lower AIC and BIC values indicate a better fit. Notably, in our study, Model 4 exhibited the lowest AIC score (50439.6) and BIC (50707.65), indicating that it offers a better fit to the data compared to the other models.

**Discussion**

There is universal consensus that secondary prevention practices such as CBE open the pathway to early detection and diagnosis of breast cancer, and in the long run, help to reduce breast cancer-related mortalities [5,40]. Yet, many women face barriers when they attempt to seek CBE. Against this background, we investigated the uptake of and barriers associated in SSA. Our findings show a low uptake of CBE among women in SSA 19.2%. The estimated prevalence of CBE uptake is supported by existing literature from SSA [35] that argues that there is a low uptake of CBE services. Additionally, Addo et al. [34] corroborate this, reporting a similarly low uptake of 16.3% among SSA women. Although these figures underscore a significant gap, there is a slight improvement noted compared to Ba et al.’s findings [36], which reported a 12% uptake. This suggests that efforts to raise awareness, improve accessibility, and enhance healthcare infrastructure may be yielding some positive results in increasing CBE uptake in SSA. This low uptake of CBE services underscores the pressing need for continued interventions and initiatives aimed at further enhancing CBE utilisation across SSA.

Our study revealed that women who had difficulty with getting permission to seek healthcare were significantly less likely to undergo screening for cervical cancer. Similar findings have been reported in a systematic review [41] that found challenges in gaining permission from husbands/partners as a major barrier to breast cancer screening. The result is also consistent with Mahajan et al. [42] findings that show lack of permission from partners as a barrier to screening uptake. Onyenwenyi and Mchunu [43] argue that in SSA countries like Nigeria, male partners do not consent for women to undergo CBE, and this stems from the fear that the woman would be examined by a male health professional. It is also imperative to understand that in many SSA countries, women may be expected to seek permission from male partners before accessing healthcare services, reflecting broader patriarchal structures that prioritize male authority. This undermines the woman’s autonomy to freely make decisions about their health and practicing preventive behaviors.

Distance also emerged as a significant barrier to the uptake of CBE services in SSA. This finding resonates with Addo et al. [34] who found a strong inverse association between women’s consideration of distance to the healthcare facility as a problem and their uptake of CBE services. The result is corroborated by Srinath et al. [41] who argued that perceived long distance to screening centres tend to be a major hurdle to women’s utilisation of CBE services. Beyond the logistical challenges posed by geographical distance [44], we propose that women’s decision-making regarding CBE uptake in SSA may be influenced by a complex cost-benefit analysis. In regions like SSA where resources are scarce and competing demands for time and financial resources are prevalent, women must weigh the perceived costs of traveling long distances to healthcare facilities against the anticipated benefits of undergoing screening [45]. This calculus extends beyond mere transportation expenses to encompass opportunity costs, such as lost wages from taking time off work or the potential need for childcare during the screening process. As such, the perceived inconvenience of travelling long distance to access CBE services may serve as a deterrent, particularly for women who face economic hardship or have limited access to information about the importance of early screening [41,45].

Consistent with extant literature from Ethiopia [46], Tanzania [47], and Ghana [48], our findings showed that women who had a big problem with getting money for healthcare were less likely to undergo screening for CBE. This is expected as CBE in the SSA countries included in this study comes at a cost. This means that women who lack the financial capacity or struggle to get financial support will find it challenging to afford the cost of screening as well as other ancillary costs such as the cost of transportation from their homes to the healthcare facility. An unexpected finding was the association between travelling alone and screening uptake. We found that women who considered travelling alone as a big problem were more likely to undergo CBE. This is surprising as it challenges the notion that autonomy is critical for improving women’s uptake of CBE services. Further research is required to fully comprehend the nuances surrounding this unexpected observation.

Beyond the main hypothesis of testing the extent to which barriers to healthcare access influence CBE uptake, we identified significant associations across the covariates. Increasing age, having higher educational attainment, being employed, having visited the healthcare facility, being exposed to the media, and being in the richest wealth index were associated with higher screening uptake – a result that aligns with a plethora of studies [34,35,36]. Additionally, women who used some sort of contraception were more likely to undergo screening. This may be explained from the perspective that women who use contraceptives are health conscious and prioritise preventive health measures.

**Implications for policy**

Considering the insights from this study, it is imperative to invest in empowering women in SSA to become more assertive and autonomous in their healthcare decision-making. Practical initiatives can include equipping women with skills to engage in economic livelihoods, drawing from successful empowerment programs in similar settings. For example, initiatives such as microfinance and vocational training programs in SSA have demonstrated significant outcomes in enhancing women's economic independence and decision-making power [49]. Addressing the financial barriers and boosting women's confidence to independently seek CBE services align with the Sustainable Development Goals (SDGs), particularly SDG 5 (Gender Equality) and SDG 3 (Good Health and Well-being). Furthermore, to address distance barriers to screening uptake, the health departments, and Ministries of Health in SSA countries should consider integrating CBE services at the primary healthcare level. This approach would bring screening services closer to communities, making them more accessible and reducing logistical challenges for women seeking care.

**Strengths and limitations**

This study relied on recent DHS data (2021/2023). Thus, our findings a reflection of the current status quo of SSA countries. Also, the large sample size coupled with the multi-stage sampling technique of the DHS ensures that the sample analyzed in this study is representative at the national and population level. The use of multi-level regression also adds to the strength of the study as it allows us to account for the different levels of factors that influence breast cancer screening uptake. Nonetheless, no causal relationship can be established due to the cross-sectional nature of the DHS. Given that we relied on secondary data, we were restricted to only variables available in the dataset. This means that other barriers (e.g., cultural norms and expectations, knowledge/information barriers, availability of facilities that provide CBE services, affordability of CBE services, healthcare provider attitudes, etc.) could not be accounted for in our model. Moreover, the study included only seven SSA countries that have available DHS datasets with a variable measuring CBE in the individual recode file (IR file), which makes us limited to extrapolating our results to all SSA countries. Similarly, our findings are limited to women of reproductive age; thus, the findings may not represent the situation in older women (50 years and older).

**Conclusion**

We conclude that barriers such as difficulties in obtaining permission, long distances to healthcare facilities, and financial constraints significantly reduce the likelihood of women undergoing CBE. The study underscores a need to improve access to healthcare facilities. Practically, this can be achieved by expanding mobile health services and integrating CBE into primary healthcare will help overcome distance-related challenges. Additionally, targeted outreach and transportation initiatives are necessary to support women facing travel barriers.

**Abbreviations**

aOR: adjusted odds ratios; CBE: Clinical breast examination; DHS: Demographic and Health Survey; SSA: sub-Saharan Africa

**Acknowledgements**

We acknowledge the Measure DHS for granting us free access to the dataset used in this study.

**Availability of data and materials**

The datasets generated and/or analysed during the current study are available in the Measure DHS repository: [http:www.//dhsprogram.com/data/available-datasets.cfm](http://dhsprogram.com/data/available-datasets.cfm).

**References**

1. Anderson BO, Ilbawi AM, El Saghir NS. Breast cancer in low and middle-income countries (LMIC s): a shifting tide in global health. The breast journal. 2015 Jan;21(1):111-8.
2. Clegg-Lamptey JN, Vanderpuye V, Dedey F. Late presentation of breast cancer in lower-and middle-income countries. Current Breast Cancer Reports. 2019 Sep 15; 11:143-51.
3. Montazeri A. Health-related quality of life in breast cancer patients: a bibliographic review of the literature from 1974 to 2007. Journal of experimental & clinical cancer research. 2008 Aug; 27:1-31.
4. Trimboli RM, Giorgi Rossi P, Battisti NM, Cozzi A, Magni V, Zanardo M, Sardanelli F. Do we still need breast cancer screening in the era of targeted therapies and precision medicine? Insights into Imaging. 2020 Dec; 11:1-0.
5. Milosevic M, Jankovic D, Milenkovic A, Stojanov D. Early diagnosis and detection of breast cancer. Technology and Health Care. 2018 Jan 1;26(4):729-59.
6. Sauter ER. Breast cancer prevention: current approaches and future directions. European journal of breast health. 2018 Apr;14(2):64.
7. Seely JM, Alhassan T. Screening for breast cancer in 2018—what should we be doing today? Current Oncology. 2018 Jun;25(s1):115-24.
8. Nelson HD, Fu R, Cantor A, Pappas M, Daeges M, Humphrey L. Effectiveness of breast cancer screening: systematic review and meta-analysis to update the 2009 US Preventive Services Task Force recommendation. Annals of internal medicine. 2016 Feb 16;164(4):244-55.
9. Iranmakani S, Mortezazadeh T, Sajadian F, Ghaziani MF, Ghafari A, Khezerloo D, Musa AE. A review of various modalities in breast imaging: technical aspects and clinical outcomes. Egyptian Journal of Radiology and Nuclear Medicine. 2020 Dec;51:1-22.
10. Helvie MA, Bevers TB. Screening mammography for average-risk women: the controversy and NCCN’s position. Journal of the National Comprehensive Cancer Network. 2018 Nov 1;16(11):1398-404.
11. Schonberg MA. Decision‐making regarding mammography screening for older women. Journal of the American Geriatrics Society. 2016 Dec;64(12):2413-8.
12. Bhan AD, Jayaram J. Screening, Self-Examination and Awareness in Breast Cancer. InBreast Cancer: Comprehensive Management 2022 Jan 31 (pp. 587-600). Singapore: Springer Nature Singapore.
13. Black E, Richmond R. Improving early detection of breast cancer in sub-Saharan Africa: why mammography may not be the way forward. Globalization and health. 2019 Dec;15:1-1.
14. Mishra GA, Pimple SA, Mittra I, Badwe RA. Screening for breast cancer: Cost-effective solutions for low-& middle-income countries. Indian Journal of Medical Research. 2021 Aug 1;154(2):229-36.
15. Cho N, Han W, Han BK, Bae MS, Ko ES, Nam SJ, Chae EY, Lee JW, Kim SH, Kang BJ, Song BJ. Breast cancer screening with mammography plus ultrasonography or magnetic resonance imaging in women 50 years or younger at diagnosis and treated with breast conservation therapy. JAMA oncology. 2017 Nov 1;3(11):1495-502.
16. Lee CH, Dershaw DD, Kopans D, Evans P, Monsees B, Monticciolo D, Brenner RJ, Bassett L, Berg W, Feig S, Hendrick E. Breast cancer screening with imaging: recommendations from the Society of Breast Imaging and the ACR on the use of mammography, breast MRI, breast ultrasound, and other technologies for the detection of clinically occult breast cancer. Journal of the American college of radiology. 2010 Jan 1;7(1):18-27.
17. Onega T, Beaber EF, Sprague BL, Barlow WE, Haas JS, Tosteson AN, D. Schnall M, Armstrong K, Schapira MM, Geller B, Weaver DL. Breast cancer screening in an era of personalized regimens: A conceptual model and National Cancer Institute initiative for risk‐based and preference‐based approaches at a population level. Cancer. 2014 Oct 1;120(19):2955-64.
18. Takkar N, Kochhar S, Garg P, Pandey AK, Dalal UR, Handa U. Screening methods (clinical breast examination and mammography) to detect breast cancer in women aged 40–49 years. Journal of mid-life health. 2017 Jan 1;8(1):2-10.
19. World Health Organization. Global breast cancer initiative implementation framework: assessing, strengthening and scaling-up of services for the early detection and management of breast cancer. World Health Organization; 2023 Mar 7.
20. American Cancer Society. 2019. *Frequently Asked Questions About the American Cancer Society’s Breast Cancer Screening Guideline*. American Cancer Society. [https://www.cancer.org/cancer/types/breast-cancer/frequently-asked-questions-about-the-american-cancer-society-new-breast-cancer-screening-guideline.html. Accessed 17 Apr 2024](https://www.cancer.org/cancer/types/breast-cancer/frequently-asked-questions-about-the-american-cancer-society-new-breast-cancer-screening-guideline.html.%20Accessed%2017%20Apr%202024).
21. Sharma R, Fronterre C, Ssentongo AE, Yenney K, Amponsah-Manu F, Ssentongo P. Mapping cancer in Africa: a comprehensive and comparable characterization of 34 cancer types using estimates from GLOBOCAN 2020. Frontiers in public health. 2022 Apr 25;10:839835.
22. Akuoko CP, Armah E, Sarpong T, Quansah DY, Amankwaa I, Boateng D. Barriers to early presentation and diagnosis of breast cancer among African women living in sub-Saharan Africa. PloS one. 2017 Feb 13;12(2):e0171024.
23. Pace LE, Shulman LN. Breast cancer in sub-Saharan Africa: challenges and opportunities to reduce mortality. The oncologist. 2016 Jun 1;21(6):739-44.
24. Anyigba CA, Awandare GA, Paemka L. Breast cancer in sub-Saharan Africa: The current state and uncertain future. Experimental Biology and Medicine. 2021 Jun;246(12):1377-87.
25. Pierz AJ, Randall TC, Castle PE, Adedimeji A, Ingabire C, Kubwimana G, Uwinkindi F, Hagenimana M, Businge L, Musabyimana F, Munyaneza A. A scoping review: Facilitators and barriers of cervical cancer screening and early diagnosis of breast cancer in Sub-Saharan African health settings. Gynecologic oncology reports. 2020 Aug 1;33:100605.
26. Formenti B, Gregori N, Crosato V, Marchese V, Tomasoni LR, Castelli F. The impact of COVID-19 on communicable and non-communicable diseases in Africa: a narrative review. Le Infezioni in Medicina. 2022;30(1):30.
27. Jailobaeva K, Falconer J, Loffreda G, Arakelyan S, Witter S, Ager A. An analysis of policy and funding priorities of global actors regarding noncommunicable disease in low-and middle-income countries. Globalization and health. 2021 Dec;17:1-5.
28. Luna F, Luyckx VA. Why have non-communicable diseases been left behind?. Asian bioethics review. 2020 Mar;12(1):5-25.
29. Nyaaba GN, Stronks K, Masana L, Larrea-Killinger C, Agyemang C. Implementing a national non-communicable disease policy in sub-Saharan Africa: Experiences of key stakeholders in Ghana. Health Policy Open. 2020 Dec 1;1:100009.
30. Shu J, Jin W. Prioritizing non-communicable diseases in the post-pandemic era based on a comprehensive analysis of the GBD 2019 from 1990 to 2019. Scientific Reports. 2023 Aug 16;13(1):13325.
31. Bray F, Parkin DM, Gnangnon F, Tshisimogo G, Peko JF, Adoubi I, Assefa M, Bojang L, Awuah B, Koulibaly M, Buziba N. Cancer in sub-Saharan Africa in 2020: a review of current estimates of the national burden, data gaps, and future needs. The Lancet Oncology. 2022 Jun 1;23(6):719-28.
32. Corsi DJ, Neuman M, Finlay JE, Subramanian SV. Demographic and health surveys: a profile. Int J Epidemiol. 2012;41(6):1602–13.
33. Obeagu EI, Obeagu GU. Breast cancer: A review of risk factors and diagnosis. Medicine. 2024 Jan 19;103(3):e36905.
34. Addo IY, Acquah E, Ayebeng C, Dickson KS. Influence of distance to health facilities on clinical breast cancer screening behaviour among women in five sub-Saharan African countries. BMC Public Health. 2023 May 19;23(1):915.
35. Afaya A, Laari TT, Seidu AA, Afaya RA, Daniels-Donkor SS, Yakong VN, Ahinkorah BO. Factors associated with the uptake of clinical breast examination among women of reproductive age in Lesotho: analysis of a national survey. BMC cancer. 2023 Feb 1;23(1):114.
36. Ba DM, Ssentongo P, Agbese E, Yang Y, Cisse R, Diakite B, Traore CB, Kamate B, Kassogue Y, Dolo G, Dembele E. Prevalence and determinants of breast cancer screening in four sub-Saharan African countries: a population-based study. BMJ open. 2020 Oct 1;10(10):e039464.
37. ICF International. Demographic and Health Survey Sampling and Household Listing Manual. MEASURE DHS, Calverton, Maryland, U.S.A.: ICF International 2012.
38. Amoah A, Issaka J, Ayebeng C, Okyere J. Influence of women empowerment on childhood (12–23 months) immunisation coverage: Recent evidence from 17 sub-Saharan African countries. Tropical Medicine and Health. 2023 Nov 14;51(1):63.
39. Vandenbroucke JP, Elm EV, Altman DG, Gøtzsche PC, Mulrow CD, Pocock SJ, Poole C, Schlesselman JJ, Egger M, Strobe Initiative. Strengthening the Reporting of Observational Studies in Epidemiology (STROBE): explanation and elaboration. Annals of internal medicine. 2007 Oct 16;147(8):W-163.
40. Grimm LJ, Avery CS, Hendrick E, Baker JA. Benefits and risks of mammography screening in women ages 40 to 49 years. Journal of primary care & community health. 2022 Jan;13:21501327211058322.
41. Srinath A, van Merode F, Rao SV, Pavlova M. Barriers to cervical cancer and breast cancer screening uptake in low-and middle-income countries: a systematic review. Health policy and planning. 2023 May 1;38(4):509-27.
42. Mahajan M, Naik N, Jain K, Patira N, Prasad S, Mogri S, Muwonge R, Lucas E, Faruq F, Sankaranarayanan R, Iyer S. Study of knowledge, attitudes, and practices toward risk factors and early detection of noncommunicable diseases among rural women in India. Journal of Global Oncology. 2019 Apr;5:1-0.
43. Onyenwenyi AO, Mchunu GG. Barriers to cervical cancer screening uptake among rural women in South West Nigeria: A qualitative study. South African Journal of Obstetrics and Gynaecology. 2018 May 31;24(1):19-23.
44. Gakunga R, Kinyanjui A, Ali Z, Ochieng’ E, Gikaara N, Maluni F, Wata D, Kyeng’ M, Korir A, Subramanian S. Identifying barriers and facilitators to breast cancer early detection and subsequent treatment engagement in Kenya: a qualitative approach. The oncologist. 2019 Dec 1;24(12):1549-56.
45. Tapera R, Senabye PK, Mhaka-Mutepfa M, January J, Apau SG. The use of the Health Belief Model (HBM) in determining the factors associated with breast cancer screening among female students in Botswana. International Journal of Health Promotion and Education. 2019 Jul 4;57(4):203-16.
46. Getachew S, Tesfaw A, Kaba M, Wienke A, Taylor L, Kantelhardt EJ, Addissie A. Perceived barriers to early diagnosis of breast Cancer in south and southwestern Ethiopia: a qualitative study. BMC women’s health. 2020 Dec;20:1-8.
47. Sakafu LL, Philipo GS, Malichewe CV, Fundikira LS, Lwakatare FA, Van Loon K, Mushi BP, DeBoer RJ, Bialous SA, Lee AY. Delayed diagnostic evaluation of symptomatic breast cancer in sub-Saharan Africa: A qualitative study of Tanzanian women. Plos one. 2022 Oct 6;17(10):e0275639.
48. Wuur MM, Duodu DA, Tarkang EE. Factors that influence breast cancer screening among women of reproductive age in the Nandom Municipality, Ghana. BMC Women’s Health. 2022 Aug 31;22(1):359.
49. Ogema EA, Bundi ZM, Mkutu A, Omollo D. The role of vocational training centers in socioeconomic empowerment of women in Nakuru Town, Kenya. International Journal of Research in Business and Social Science (2147-4478). 2021 May 1;10(3):380-90.

# **Supporting Information**

S1 File STROBE Checklist

**Table 1: Prevalence of CBE among women by countries**

| **Country** | **Survey year** | **Weighted sample** | **Percentage of women who attended CBE [95%CI]** |
| --- | --- | --- | --- |
| Burkina Faso | 2021 | 10,550 | 31.1[29.1-33.1] |
| Cote d’Ivoire | 2021 | 8,959 | 17.0[15.3-18.9] |
| Ghana | 2022 | 9,639 | 22.5[21.0-24.2] |
| Kenya | 2022 | 10,494 | 18.5[17.4-19.6] |
| Mozambique | 2022-23 | 6,977 | 11.2[9.9-12.6] |
| Senegal | 2023 | 9,455 | 22.9[21.1-24.9] |
| Tanzania | 2022 | 9,413 | 7.3[6.5-8.2] |
| **All countries** |  | **65,486** | **19.2[18.5-19.8]** |

**#**All percentages are calculated in row percentages

**Table 2: Distribution of clinical breast examination across identified barriers by country**

| **Barriers** | **Clinical Breast Examination (CBE)** | | | | | | | | | | | | | |
| --- | --- | --- | --- | --- | --- | --- | --- | --- | --- | --- | --- | --- | --- | --- |
|  | **Burkina Faso** | | **Cote d’Ivoire** | | **Ghana** | | **Kenya** | | **Mozambique** | | **Senegal** | | **Tanzania** | |
|  | **[n(%)]** | p-value | **[n(%)]** | p-value | **[n(%)]** | p-value | **[n(%)]** | p-value | **[n(%)]** | p-value | **[n(%)]** | p-value | **[n(%)]** | p-value |
| **Permission to visit health facility** |  | **<0.001** |  | **<0.001** |  | **<0.001** |  | 0.093 |  | **0.014** |  | 0.574 |  | 0.557 |
| Big problem | 365(24.0) |  | 412(12.4) |  | 149(17.1) |  | 91(17.9) |  | 33(5.5) |  | 566(23.3) |  | 40(6.4) |  |
| Not a big problem | 2,922(32.2) |  | 1,150(19.7) |  | 1,994(23.1) |  | 1,845(18.5) |  | 764(11.6) |  | 1,566(22.8) |  | 643(7.3) |  |
| **Money needed for treatment** |  | **<0.001** |  | **<0.001** |  | **<0.001** |  | **<0.001** |  | **<0.001** |  | **0.006** |  | **<0.001** |
| Big problem | 1,900(27.9) |  | 851(14.5) |  | 762(17.4) |  | 822(16.3) |  | 137(5.6) |  | 1,365(22.4) |  | 191(5.5) |  |
| Not a big problem | 1,386(36.9) |  | 711(21.6) |  | 1,381(26.9) |  | 1,114(20.5) |  | 660(14.2) |  | 767(24.1) |  | 491(8.4) |  |
| **Distance to a health facility** |  | **<0.001** |  | **<0.001** |  | **<0.001** |  | **<0.001** |  | **<0.001** |  | 0.774 |  | **<0.001** |
| Big problem | 1,062(26.8) |  | 541(13.7) |  | 343(15.8) |  | 403(15.5) |  | 152(5.4) |  | 801(24.0) |  | 135(4.9) |  |
| Not a big problem | 2,225(33.6) |  | 1,021(19.5) |  | 1,800(24.8) |  | 1,532(19.5) |  | 644(15.0) |  | 1,332(22.4) |  | 548(8.3) |  |
| **Travel alone** |  | 0.451 |  | **<0.001** |  | **<0.001** |  | **0.009** |  | **0.002** |  | 0.284 |  | **0.014** |
| Big problem | 514(30.5) |  | 290(11.9) |  | 260(18.8) |  | 146(17.0) |  | 53(8.0) |  | 542(25.5) |  | 88(6.2) |  |
| Not a big problem | 2,773(31.2) |  | 1,272(18.9) |  | 1,883(23.2) |  | 2,352(18.6) |  | 744(11.6) |  | 1,591(22.2) |  | 595(7.5) |  |
| **At least one barrier** |  | **<0.001** |  | **<0.001** |  | **<0.001** |  | **<0.001** |  | **<0.001** |  | **0.006** |  | **<0.001** |
| Big problem | 2,122(28.8) |  | 952(14.7) |  | 924(18.1) |  | 943(16.8) |  | 239(6.9) |  | 1,467(22.3) |  | 263(5.6) |  |
| Not a big problem | 1,165(36.4) |  | 611(22.7) |  | 1,219(27.8) |  | 992(20.5) |  | 557(15.4) |  | 665(24.4) |  | 419(9.0) |  |

**#**All percentages are calculated in row percentages

**Table 3: Proportion of CBE utilisation among women by selected covariates**

| **Covariates** | **Women who screened for CBE** | | **p-value** |
| --- | --- | --- | --- |
|  | **Frequency (n)** | **Percent (%)[95%CI]** |  |
| **Age** |  |  | <0.001 |
| 25-29 years | 3,033 | 17.6[16.8-18.5] |  |
| 30-34 years | 2,967 | 19.5[18.5-20.5] |  |
| 35-39 years | 2,876 | 20.6[19.6-21.7] |  |
| 40-44 years | 2,129 | 19.7[18.7-20.8] |  |
| 45-49 years | 1,550 | 18.7[17.5-19.9] |  |
| **Education** |  |  | <0.001 |
| No education | 4,245 | 16.8[15.9-17.8] |  |
| Primary | 2,630 | 13.5[12.8-14.3] |  |
| Secondary | 3,671 | 23.6[22.5-24.7] |  |
| higher | 2,008 | 38.5[36.4-40.6] |  |
| **Occupational status** |  |  | <0.001 |
| Working | 8,849 | 20.7[20.0-21.5] |  |
| Not working | 3,707 | 16.2[15.3-17.2] |  |
| **Marital status** |  |  | <0.001 |
| Never married | 1,059 | 20.1[18.5-21.7] |  |
| Married | 10,127 | 19.5[18.8-20.2] |  |
| Formerly married | 1,369 | 16.8[15.6-18.0] |  |
| **Religion** |  |  | <0.001 |
| Muslim | 5,195 | 21.9[20.8-23.3] |  |
| Christian | 6,457 | 21.5[20.7-22.3] |  |
| Other | 227 | 9.6[8.1-11.2] |  |
| **Wealth index** |  |  | <0.001 |
| Poorest | 1,167 | 10.5[9.6-11.5] |  |
| Poorer | 1,603 | 14.0[13.0-15.1] |  |
| Middle | 2,045 | 16.4[15.3-17.5] |  |
| Richer | 2,831 | 20.0[18.9-21.2] |  |
| Richest | 4,909 | 30.0[28.8-31.3] |  |
| **Visit health facility in last 12 months** |  |  | <0.001 |
| No | 3,339 | 13.3[12.6-14.0] |  |
| Yes | 9,217 | 22.9[22.1-23.7] |  |
| **Contraceptive usage** |  |  | <0.001 |
| Not using | 7,434 | 17.5[16.9-18.3] |  |
| Modern contraceptive | 4,354 | 21.7[20.9-22.7] |  |
| Traditional method | 768 | 24.6[22.6-26.8] |  |
| **Media exposure within a week** |  |  | <0.001 |
| Exposed | 10,968 | 21.9[21.2-22.6] |  |
| unexposed | 1,587 | 10.3[9.5-11.2] |  |
| **Type of residence** |  |  | <0.001 |
| Urban | 7,129 | 24.5[23.5-25.5] |  |
| Rural | 5,426 | 14.9[14.1-15.7] |  |

**#**All percentages are calculated in row percentages

**Table 4: Multilevel logistic regression analysis of barriers associated with CBE in Seven SSA countries**

| **Explanatory variables** | **Null model (0)** | **Model I** | **Model II** | **Model III** | **Model IV (full model)** |
| --- | --- | --- | --- | --- | --- |
|  |  | aOR(95%CI) | aOR(95%CI) | aOR(95%CI) | aOR(95%CI) |
| ***Fixed effect:*** |  |  |  |  |  |
| **Permission** |  |  |  |  |  |
| Not a problem |  | 1.00 | 1.00 | 1.00 | 1.00 |
| A big problem |  | 0.98[0.91-1.05] | 0.88***[0.81-0.94] | 0.90**[0.84-0.97] | 0.88**[0.82-0.95] |
| **Money** |  |  |  |  |  |
| Not a problem |  | 1.00 | 1.00 | 1.00 | 1.00 |
| A big problem |  | 0.89***[0.85-0.94] | 1.03[0.98-1.08] | 0.75***[0.75-0.79] | 0.92**[0.88-0.97] |
| **Distance** |  |  |  |  |  |
| Not a problem |  | 1.00 | 1.00 | 1.00 | 1.00 |
| A big problem |  | 0.70***[0.67-0.74] | 0.90***[0.84-0.95] | 0.86***[0.81-0.91] | 0.95*[0.89-0.99] |
| **Travel alone** |  |  |  |  |  |
| Not a problem |  | 1.00 | 1.00 | 1.00 | 1.00 |
| A big problem |  | 1.13***[1.06-1.21] | 1.21***[1.13-1.30] | 1.14***[1.07-1.23] | 1.19***[1.10-1.28] |
| **Age (years)** |  |  |  |  |  |
| 25-29 |  |  | 1.00 |  | 1.00 |
| 30-34 |  |  | 1.15***[1.08-1.22] |  | 1.17***[1.10-1.25] |
| 35-39 |  |  | 1.25***[1.17-1.34] |  | 1.31***[1.22-1.40] |
| 40-44 |  |  | 1.24***[1.16-1.33] |  | 1.29***[1.20-1.39] |
| 45-49 |  |  | 1.20***[1.11-1.30] |  | 1.26***[1.17-1.37] |
| **Education** |  |  |  |  |  |
| No education |  |  | 0.55***[0.50-0.60] |  | 0.37***[0.33-0.41] |
| Primary |  |  | 0.46***[0.43-0.51] |  | 0.43***[0.39-0.47] |
| Secondary |  |  | 0.68***[0.62-0.73] |  | 0.59***[0.54-0.64] |
| Higher |  |  | 1.00 |  | 1.00 |
| **Occupational status** |  |  |  |  |  |
| Working |  |  | 1.00 |  | 1.00 |
| Not working |  |  | 0.84***[0.80-0.89] |  | 0.91***[0.86-0.95] |
| **Religion** |  |  |  |  |  |
| Other |  |  | 1.00 |  | 1.00 |
| Muslim |  |  | 2.15***[1.87-2.47] |  | 1.56***[1.34-1.80] |
| Christian |  |  | 1.63***[1.41-1.87] |  | 1.81***[1.57-2.09] |
| **Visit health facility in last 12 months** | |  |  |  |  |
| Yes |  |  | 1.00 |  | 1.00 |
| No |  |  | 0.58***[0.55-0.61] |  | 0.62***[0.59-0.66] |
| **Wealth index** |  |  |  |  |  |
| Poorest |  |  | 1.00 |  | 1.00 |
| Poorer |  |  | 1.23***[1.13-1.33] |  | 1.16***[1.07-1.26] |
| Middle |  |  | 1.40***[1.29-1.51] |  | 1.28***[1.17-1.39] |
| Richer |  |  | 1.62***[1.49-1.76] |  | 1.42***[1.30-1.56] |
| Richest |  |  | 2.55***[2.34-2.77] |  | 2.01***[1.81-2.23] |
| **Contraceptive usage** | |  |  |  |  |
| Not using |  |  | 1.00 |  | 1.00 |
| Modern contraceptive |  |  | 1.20***[1.15-1.26] |  | 1.20***[1.14-1.27] |
| Traditional method | |  | 1.31***[1.18-1.46] |  | 1.32***[1.19-1.47] |
| **Media exposure within a week** | |  |  |  |  |
| Exposed |  |  | 1.00 |  | 1.00 |
| Unexposed |  |  | 0.71***[0.67-0.76] |  | 0.75***[0.70-0.80] |
| ***Community-level factors*** | |  |  |  |  |
| **Type of residence** | |  |  |  |  |
| Urban |  |  |  | 1.00 | 1.00 |
| Rural |  |  |  | 0.53***[0.50-0.51] | 0.88***[0.83-0.94] |
| **Country** |  |  |  |  |  |
| Burkina Faso |  |  |  | 1.00 | 1.00 |
| Cote d’Ivoire |  |  |  | 0.32***[0.30-0.35] | 0.37***[0.33-0.39] |
| Ghana |  |  |  | 0.45***[0.42-0.48] | 0.38***[0.34-0.40] |
| Kenya |  |  |  | 0.33***[0.30-0.35] | 0.24***[0.20-0.24] |
| Mozambique |  |  |  | 0.27***[0.25-0.29] | 0.23***[0.21-0.25] |
| Senegal |  |  |  | 0.55***[0.55-0.63] | 0.66***[0.61-0.72] |
| Tanzania |  |  |  | 0.13***[0.12-0.15] | 0.***[0.78-0.91] |
| ***Random-effect model*** | |  |  |  |  |
| Community variance (SE) | 0.152(0.01) | 0.146(0.01) | 0.142(0.01) | 0.166(0.02) | 0.196(0.01) |
| PCV (%) | 1.00 | -.1.15 | 7.0 | 16.9 | 18.1 |
| ICC (%) | 4.4 | 4.2 | 4.1 | 4.6 | 4.7 |
| ***Model fit statistics*** | |  |  |  |  |
| Log-likelihood | -30475.116 | -30335.514 | -25923.555 | -28759.601 | -25189.799 |
| AIC | 60954.23 | 60683.03 | 51895.11 | 57537.2 | 50439.6 |
| BIC | 60972.41 | 60737.57 | 52109.55 | 57619.01 | 50707.65 |

1.00: reference category; aOR: adjusted odds ratio; 95%CI: 95% confidence interval; ICC: Intraclass correlation; PCV: Proportional change in variance; AIC: Akaike information criterion; BIC: Bayesian information criterion

*p<0.05, **p<0.01, ***p<0.001
